# TRPM8 Channels and Dry Eye

**DOI:** 10.3390/ph11040125

**Published:** 2018-11-15

**Authors:** Jee Myung Yang, Edward T. Wei, Seong Jin Kim, Kyung Chul Yoon

**Affiliations:** 1 Department of Ophthalmology, Chonnam National University Medical School and Hospital, Gwangju 61469, Korea; jeemang87@naver.com; 2 Graduate School of Medical Science and Engineering, Korea Advanced Institute of Science and Technology, Daejeon 34141, Korea; 3 School of Public Health, University of California, Berkeley, CA 94720, USA; koolicin@yahoo.com; 4 Department of Dermatology, Chonnam National University Medical School and Hospital, Gwangju 61469, Korea; seongkim@chonnam.ac.kr

**Keywords:** transient receptor potential, dry eye disease, TRPV, TRPM8

## Abstract

Transient receptor potential (TRP) channels transduce signals of chemical irritation and temperature change from the ocular surface to the brain. Dry eye disease (DED) is a multifactorial disorder wherein the eyes react to trivial stimuli with abnormal sensations, such as dryness, blurring, presence of foreign body, discomfort, irritation, and pain. There is increasing evidence of TRP channel dysfunction (i.e., TRPV1 and TRPM8) in DED pathophysiology. Here, we review some of this literature and discuss one strategy on how to manage DED using a TRPM8 agonist.

## 1. Emerging Concepts of Neural–Sensory Mechanisms in Dry Eye

Dry eye disease (DED) is a multifactorial disorder of the ocular surface, and especially of the sensory and motor nerves that regulate the physiology of this surface [1]. It is an economic burden to society, as it affects 5–30% of the population in a wide range of age groups [2]. Considering the pathophysiology of DED, the treatment strategy has shifted from just hydrating and lubricating the ocular surface to modifying the underlying disease process. Traditionally, aqueous tear deficiency is considered one of the major symptoms of DED, which is caused by a deficit of lacrimal and conjunctival tear secretion [3]. Recently, increased attention has focused on the neuronal regulation of glandular tear secretion [3,4,5]. Studies also show that thermal changes at the ocular surface activate cool neurons and may affect surface wetness [6,7,8].

## 2. TRP Channels Related to Cooling Sensation in DED

**Figure 1:**
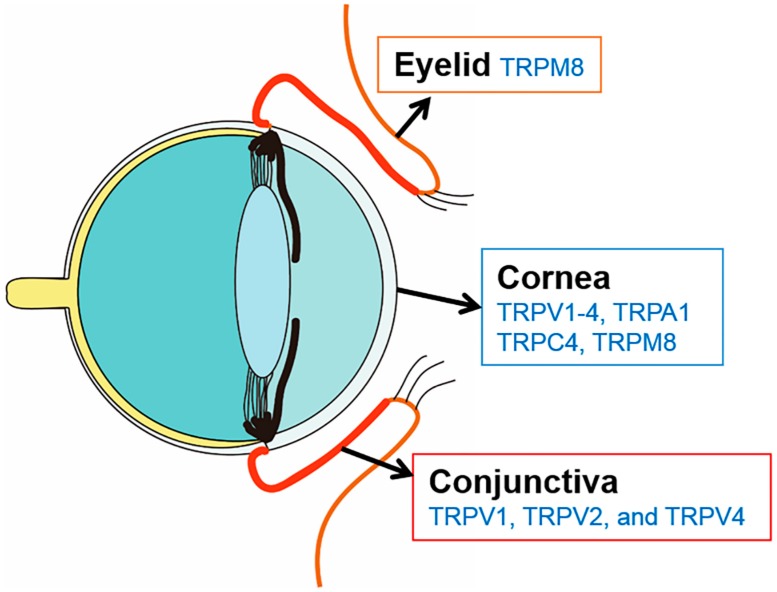
Illustration depicting identified transient receptor potential (TRP) channels in the human anterior surface.

Transient receptor potential (TRP) cation channels are associated with the perception of chemical irritation and temperature change [9]. These channels are classified into six subfamilies: (1) TRPC (canonical); (2) TRPV (vanilloid); (3) TRPM (melastatin); (4) TRPP (polycystin); (5) TRPML (mucolipin), and (6) TRPA (ankyrin) [10]. For the ocular surface, TRP channels have been identified in the cornea (TRPV1-4, TRPA1, TRPC4, and TRPM8), in the conjunctiva (TRPV1, TRPV2, and TRPV4), and in the eyelid TRPM8 (Figure 1) [10,11,12,13]. In addition, TRP receptors are differentially expressed in the corneal epithelium (TRPV1, TRPV3, TRPV4, TRPM8, and TRPC4), stroma (TRPV1, and TRPM8), and endothelium (TRPV1, TRPM8, and TRPA1) [11,14]. Within the TRP family, TRPM8 is a cold-sensing receptor (cold thermoreceptor), with a threshold of ~25 °C, and located on nerve endings of the ophthalmic branch of the trigeminal nerve [8]. TRPM8 receptors appear to be first activated on the ocular surface after evaporation of the tear film [15]. The receptor is highly sensitive to dynamic temperature reduction and is also stimulated by cooling agents, such as menthol and icilin [16]. These cold-sensitive ocular thermoreceptors are the only TRP receptors that exhibit tonic, spontaneous activity. The discharge of these afferents may regulate basal tear secretion by sensing eye wetness [17].

Dysfunction of TRPM8-mediated sensing of evaporation-induced temperature and osmolarity change in the corneal surface has been suggested as a possible pathophysiological mechanism in DED [15]. TRPM8 knock-out mice showed a reduction in basal tear secretion [8]. Corcoran et al. [18] showed that perception of cold was modified in patients with DED compared to healthy subjects. The altered sensitivity was not seen in corneal mechanoreceptors. Recently, Alcalde et al. [19] found in studies on mice that aging impairs activity of high-threshold cold thermoreceptors, which makes the ocular surface more sensitive to stimuli and ocular irritation, and to tearing. These findings substantiate the hyperactivity of the cold thermoreceptor on the cornea as one of the risk factors that cause abnormal lacrimation and contribute to the high incidence of DED in aged people. It is not clear if the manifestations of changes seen in temperature sensitivity of DED patients are triggered by the disease process or just epiphenomenon. The consensus view at this time is that abnormal TRPM8 reactivity on the cornea triggers irritation.

## 3. TRP Channels Related to Ocular Pain in DED

According to a TFOS DEWS II report, the revised definition of DED included neurosensory abnormalities emphasizing the importance of neural regulation of tearing as well as pain sensing [1,15]. Researchers focus on corneal nociceptors (polymodal nociceptors) as initiators of DED pathophysiology [15,20]. The coding of sensory neural circuits has been intensively studied [21]. The distributions and morphological specialization related to the function of TRPV1, and TRPM8 ion channels on the cornea have been dissected [22]. For example, Alamri et al. [22] showed that corneal polymodal nociceptors have TRPV1-positive small ramifying nerve endings, whereas the cold thermoreceptor has TRPM8-positive large complex endings. TRPV1 has also been detected in human corneal epithelial and conjunctival cells [13,23]. Generally, it is known that TRPV1 plays a role in DED, since it can be activated by hypertonic challenge, which in turn leads to an increased release of pro-inflammatory cytokines [24,25]. In addition, the functional transduction of the heat, irritation, and pain signal from the ocular surface has been validated for TRPV1 [21,26]. Polymodal nociceptors in the eye that preferentially contain neuropeptides are expressed through a TRPV1 channel, and those without neuropeptides are expressed through a TRPA1 channel [8,20,27,28]. These two channels are considered major detectors of external irritants, endogenous mediators, and heat on ocular surface [29]. In a murine dry eye model, the TRPV1 channel plays a major role in hypertonic saline-induced nocifensive behavior, while the TRPM8 channel is less important [30]. TRPM8 immunofluorescence is dense in the cornea and eyelid skin, but not on the conjunctiva [12,26].

## 4. Modulation of TRP Channels in DED

The cornea and conjunctiva of DED patients display abnormal hypersensitivity to normally harmless cold stimuli (cold allodynia) [31]. In this context, the use of TRPM8 antagonists could be a strategy of treatment. The utility of cooling for relieving dysesthesia and pain in DED is uncertain. In studies on humans, cooling relieved post-cataract surgery pain and cooled-artificial tears provided relief by decreasing corneal and conjunctival sensation measured by esthesiometry [32,33]. In addition, an ice pack applied to the orbit reduced the pain of injury, suggesting that TRPM8 activation is beneficial for discomfort [33]. For confirmation of the benefits of cooling, it must be accurately stated how a TRPM8 agonist applied to the ocular surface will affect sensation or discomfort in patients with DED. In animal models of DED, menthol, a TRPM8 agonist, was shown to accentuate the cooling sensation [17]. However, TRPM8 agonists, such as menthol and icilin, have limited value in ocular studies in humans. After a brief episode of cooling, menthol vapors irritated the eye and menthol solutions caused significant discomfort in patients [34]. Icilin, a more potent TRPM8 agonist than menthol, is not soluble in any ophthalmic vehicles and is, hence, difficult to deliver to target receptors [12,35].

Borneol, a bicyclic monoterpenoid compound widely used in traditional Chinese medicine, has been introduced as a TRPM8 agonist that could be used in DED treatment by increasing corneal wetness in a temperature- and dose-dependent manner [36]. By studying human conjunctival epithelial cells, researchers found that thyronamine, an endogenous thyroid hormone metabolite, activated the TRPM8 channel and prevented the capsaicin-induced activation of the TRPV1 channel [37]. In addition, the effect of thyronamine has been validated in human corneal epithelial cells, suggesting this molecule as a novel endogenous modulator of TRPM8 in the ocular surface [38,39]. The antagonism of TRPM8 by *N*-(3-aminopropyl)-2-{[(3-methylphenyl)methyl]oxy}-*N*-(2-thienylmethyl) benzamide (AMTB) has also been considered for DED treatment, since evaporative cooling and hyperosmotic stimuli trigger dry eye pain as well as blinking [40]. However, an undesirable side effect of antagonists may be the reduction of tear secretion [8]. For example, the ocular application of the TRPM8 antagonist (*N*-(4-tertiarybutylphenyl)-4-(3-chloro-pyridin-2-yl)-tetrahydropyrazine-1(2*H*)-carbox-amide) (BCTC) decreases the response to corneal dryness by 45–80% [4]. An experimental human study with systemic dosing of a selective TRPM8 antagonist [(*R*)-3-[(1-(4-fluorophenyl)ethyl)(quinolin-3-ylcarbonyl)amino] methylbenzoic acid] found no ocular symptoms when given to 22 volunteers [41]. Other strategies for modulating DED via TRP channels have been described and await proof of concept in clinical studies [42,43,44].

## 5. Novel Application of a TRPM8 Agonist in DED

**Figure 2:**
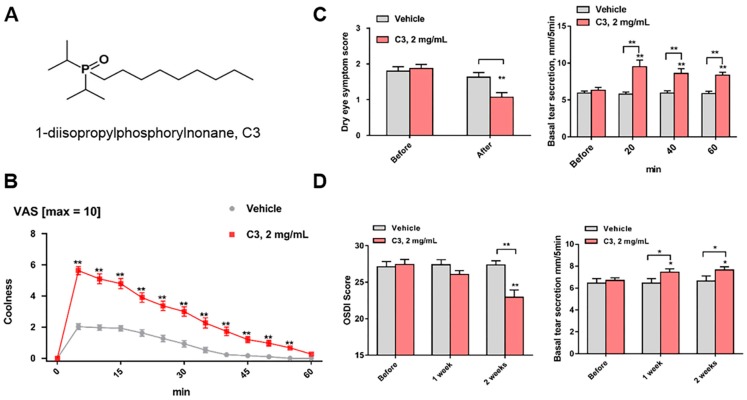
Chemical structure and function of C3. (**A**) Structure of 1-diisopropylphosphorylnonane, cryosim-3 (C3). (**B**,**C**) Visual analogue scale (VAS), (**B**) dry eye symptom score, and basal tear secretion (**C**) after single application of vehicle or C3. (**D**) Ocular surface disease index (OSDI) score, and basal tear secretion after application of vehicle of C3 four times a day for 2 weeks. * P < 0.05, ** P < 0.01, compared to baseline value or vehicle (Adapted from Yang et al. [12]).

The dialkylphosphorylalkane (Dapa) cooling agents, first described in 1978 [36], are attractive for ocular applications because some of these analogs are soluble in water at 0.5 to 5 mg/mL and provide refreshing sensations of cooling. A new Dapa TRPM8 agonist that relieves signs and symptoms of DED was described [12]. The chemical is 1-diisopropylphosphorylnonane (CAS Registry Number 1503744–37–8-7), which is called cryosim-3 (C3) (Figure 2A). C3 is an ideal candidate since it has high selectivity for TRPM8, no overt irritation, and has an optimal duration of drug action on the ocular surface. Topical administration of C3 to the closed eyelids by wiping quickly induced coolness on the periocular surface, and the cooling sensation lasted for more than 40 min by a single application (Figure 2B). C3 improves the symptoms of DED and basal tear secretion significantly without any adverse effects, such as ocular irritation or pain (Figure 2C,D). Corneal sensitivity measured by Cochet–Bonnet esthesiometry was not affected by application of 2 mg/mL solution of a related analog, 1-di-sec-butylphosphorylpentane, onto the closed eyelid by aerosol spray [unpublished data]. In addition, 5 μM of this analog did not inhibit hNav1.7 (sodium channels) in vitro, indicating the absence of lidocaine-like anesthetic activity. The method of drug delivery, that of wiping ~20 μL of solution per eye to the ocular margins where TRPM8 is expressed, avoids stimulation of the corneal polymodal neurons [20,45]. Minimizing bolus contact with the corneal nociceptors that cause sting, irritation, and pain is a critical factor that leads to the success of the wiping method of TRPM8 agonist delivery.

## 6. Conclusions

The treatment of DED becomes more challenging as people are frequently exposed to high evaporative environments. Working in front of the computer decreases the blinking rate, thereby increasing tear evaporation, which may cause dry eye-related symptoms, such as computer vision syndrome. TRP channels can be activated by trivial stresses that we encounter in daily life. So far, the translation of research findings for DED treatment has not been clearly defined in the field of TRP channels. It would be interesting to know whether TRP expressing sensory nerves and their function is preserved in patients with DED or DED-related conditions, and whether TRP modulation has the potential to treat DED in such patients. The manipulation of these TRP channels on the ocular surface may provide novel options for treating DED, especially to those refractory to conventional strategies of surface lubrication and anti-inflammatory agents. We expect to further elucidate the C3 therapeutic strategy in DED patients, including patients with Sjogren’s syndrome and neuropathic pain.
